# Tumor-Infiltrating T Cells in Skin Basal Cell Carcinomas and Squamous Cell Carcinomas: Global Th1 Preponderance with Th17 Enrichment—A Cross-Sectional Study

**DOI:** 10.3390/cells13110964

**Published:** 2024-06-03

**Authors:** Daniela Cunha, Marco Neves, Daniela Silva, Ana Rita Silvestre, Paula Borralho Nunes, Fernando Arrobas, Julie C. Ribot, Fernando Ferreira, Luís F. Moita, Luís Soares-de-Almeida, João Maia Silva, Paulo Filipe, João Ferreira

**Affiliations:** 1 Instituto de Medicina Molecular João Lobo Antunes, Faculdade de Medicina da Universidade de Lisboa, 1649-028 Lisbon, Portugal; daniela.cunha@fundacaochampalimaud.pt (D.C.);; 2 Centro de Dermatologia, Hospital CUF Descobertas, 1998-018 Lisbon, Portugal; 3 Dermatology Unit, Champalimaud Foundation, 1400-038 Lisbon, Portugal; 4 Serviço de Anatomia Patológica, Hospital CUF Descobertas, 1998-018 Lisbon, Portugalpaula.b.nunes@cuf.pt (P.B.N.); 5 Instituto de Anatomia Patológica, Faculdade de Medicina da Universidade de Lisboa, 1649-028 Lisbon, Portugal; 6 Datamedica, Biostatistics Services and Consulting, 2610-008 Amadora, Portugal; 7 CIISA—Centre for Interdisciplinary Research in Animal Health, Faculty of Veterinary Medicine, University of Lisbon, 1300-477 Lisbon, Portugal; fernandof@fmv.ulisboa.pt; 8 Associate Laboratory for Animal and Veterinary Sciences (AL4AnimalS), 1300-477 Lisbon, Portugal; 9 Innate Immunity and Inflammation Laboratory, Instituto Gulbenkian de Ciência, 2780-156 Oeiras, Portugal; 10 Serviço de Dermatologia, Centro Hospitalar Universitário Lisboa Norte EPE, 1649-028 Lisbon, Portugal; 11 Clínica Dermatológica Universitária, Faculdade de Medicina da Universidade de Lisboa, 1649-028 Lisbon, Portugal

**Keywords:** basal cell carcinoma, squamous cell carcinoma, tumor-infiltrating lymphocytes, Th1 cells, Th2 cells, Th17 cells

## Abstract

Basal cell carcinomas (BCCs) and squamous cell carcinomas (SCCs) are high-incidence, non-melanoma skin cancers (NMSCs). The success of immune-targeted therapies in advanced NMSCs led us to anticipate that NMSCs harbored significant populations of tumor-infiltrating lymphocytes with potential anti-tumor activity. The main aim of this study was to characterize T cells infiltrating NMSCs. Flow cytometry and immunohistochemistry were used to assess, respectively, the proportions and densities of T cell subpopulations in BCCs (n = 118), SCCs (n = 33), and normal skin (NS, n = 30). CD8+ T cells, CD4+ T cell subsets, namely, Th1, Th2, Th17, Th9, and regulatory T cells (Tregs), CD8+ and CD4+ memory T cells, and γδ T cells were compared between NMSCs and NS samples. Remarkably, both BCCs and SCCs featured a significantly higher Th1/Th2 ratio (~four-fold) and an enrichment for Th17 cells. NMSCs also showed a significant enrichment for IFN-γ-producing CD8+T cells, and a depletion of γδ T cells. Using immunohistochemistry, NMSCs featured denser T cell infiltrates (CD4+, CD8+, and Tregs) than NS. Overall, these data favor a Th1-predominant response in BCCs and SCCs, providing support for immune-based treatments in NMSCs. Th17-mediated inflammation may play a role in the progression of NMSCs and thus become a potential therapeutic target in NMSCs.

## 1. Introduction

Squamous cell carcinomas (SCCs) and basal cell carcinomas (BCCs) are non-melanoma skin cancers (NMSCs) comprising the most common cancers in humans [1,2,3].

Ultraviolet (UV) radiation is a major carcinogen in skin cancer and, consequently, BCCs and SCCs are commonly located on sun-exposed areas, such as the face [4,5,6,7].

NMSC are classified in high- vs. low-risk groups according to the anatomic site, size, patient immune status, primary vs. recurrent, histological subtype, and other histopathologic criteria [5,6]. Although the risk of metastasis is low for both BCCs (<0.1% [5]) and SCCs (1.2–5% [8,9]), high-risk tumors may become locally destructive due to the extensive involvement of skin and underlying structures. Locally aggressive and deep penetrating BCCs and SCCs of the face may ultimately lead to disfigurement and functional impairment [7].

In onco-immunology, it is recognized that initial phases of immune surveillance and equilibrium are followed by a phase of escape, in which the cancer cells acquire the ability to evade the immune surveillance [10,11,12]. Tumor-infiltrating lymphocytes (TILs) are common constituents of the tumor microenvironment (TME) and, by providing bioactive molecules to the TME, play a major role in cancer immunity [13,14,15], influencing tumor progression and prognosis [16,17]. Indeed, over the last decade, the success of immunotherapy in various cancers has highlighted the role of TILs in cancer [18,19].

TILs include CD4+ and CD8+ T cells. CD8+ T cells are ubiquitous components of the TME and can differentiate into cytotoxic T lymphocytes (CTLs), which play an important role in the anti-cancer host response, with functional relevance for cancer immunotherapy [2,19,20,21,22,23,24,25,26]. CD4+ T cells can polarize into Th1 (T helper type 1) or Th2 (T helper type 2) cells, along with other subtypes. By secreting IL-2, TNFα, and IFN-γ, Th1-polarized CD4+ T cells play a relevant role in anti-tumor immunity [27,28,29,30], including in NMSCs [31,32,33], and may predicate the success of anti-cancer immune therapy [34,35]. Conversely, by producing IL-4, -5, and -13, Th2-polarized CD4+ T cells may be involved in the downregulation of T cell-mediated cytotoxicity and in the induction of a tumor-promoting response [29,36,37].

Some past studies have analyzed the composition of the cytokine milieu within the TME of NMSCs, leading to the conclusion of a mostly immunosuppressive Th2- and regulatory T (Treg) cell-favorable TME, considering the relative enrichment of IL-4, IL-10, and CCL22 [2,21,38,39,40]. However, the relative balance between Th1 and Th2 cells in NMSCs still remains largely unaddressed.

Regulatory T (Treg) cells are a population of CD4+ T cells defined by their expression of forkhead winged-helix transcription factor (FoxP3). Due to their immunosuppressive effects, Tregs may negatively influence anti-tumor immunity [41,42,43] and, in general, a higher proportion of Tregs is associated with a worse prognosis [44]. Furthermore, tumors harboring a decreased ratio between CD8+ cells and Treg cells (CD8+/Treg ratio) are mostly associated with a worse prognosis [45,46,47]. With respect to BCCs and SCCs, both tumor types have been shown to harbor a high density of Treg cells [21,48,49]. Whether the percentage of Tregs within the CD4+ T cell population is increased in NMSCs relative to control normal skin (NS) is, however, not consensual [21,36,40,48,49]. A decreased CD8+/Treg ratio has been observed in aggressive subtypes of SCC, suggesting a correlation between this ratio and SCC aggressiveness [50].

Several other T cell subpopulations, such as gamma-delta (γδ), Th17, and Th9 cells, infiltrate tumors and influence anti-tumor immunity [51,52,53,54]. γδ T cells have been assigned a critical role in cancer immunosurveillance [51,55] and have, therefore, become a hot topic in cancer therapy [56,57,58]. However, recent data strongly suggest that γδ T cells may also polarize toward a pro-tumor phenotype depending on the cytokines present in the TME [59].

The potential role of Th17 cells in cancer has recently attracted much attention since this T cell population has been found in several tumors [60]. In NMSC, IL-17- and IL-22-producing lymphocytes have been identified in peritumoral skin, and in vitro and in vivo pre-clinical studies have suggested that IL-17 can promote tumor progression [61,62,63]. Although Th17 cells have been implicated in the host reaction to tumors, the extent of their involvement in NMSC has remained poorly characterized [33,61,62,63].

Th9 cells are a recently described subpopulation of CD4+ T cells exhibiting controversial effects in cancer immunity, depending on the tumor type [54,64,65].

The success of immune-targeted therapies in advanced NMSCs in recent years [66,67,68,69,70,71,72] led us to hypothesize that NMSCs may harbor significant populations of TILs, with a potential to drive anti-tumor activities. Since a clear picture of the composition of TILs in NMSCs is still missing, this study aimed at identifying subpopulations of T cells that infiltrate the tumor microenvironment of BCCs and SCCs, and at clarifying whether the balance between these subpopulations differed from normal skin. To this end, we studied T cell subpopulations, such as CD4+ and CD8+ T cells, Th1 and Th2 cells, and Treg, Th17, Th9, and γδ cells, by combining flow cytometry and immunohistochemistry (IHC). This dual approach provides enhanced analytical power since flow cytometry excels at providing accurate proportions between T cell subpopulations, while IHC yields better estimates of population densities and distribution (cells per area).

## 2. Materials and Methods

Patients were recruited at the Dermatology Outpatient Clinic of Centro Hospitalar de Lisboa Norte and Hospital CUF Descobertas, in Lisbon. The tumor samples were consecutive cases of BCC and SCC referred for surgical treatment according to the National Comprehensive Cancer Network guidelines [5,6]. Excised tumors were examined by a certified histopathologist. Normal skin (NS) samples were Burrow’s triangles obtained upon removal of melanocytic nevi from healthy donors. Exclusion criteria were as follows: (a) age under 18 years old, (b) pregnant women, (c) patients undergoing chemotherapy, (d) patients with known lymphoid disorders, and (e) lesions subjected to topical treatment in the previous 12 weeks. Clinical data (gender, age, and tumor site) were recorded at initial evaluation. The study was approved by the Local Ethics Committees and was conducted according to the Declaration of Helsinki Principles.

### 2.1. Tissue Processing and Stimulation of Cytokine Production

BCC (n = 118) and SCC (n = 33) samples collected from the center of the tumors with a 4 mm biopsy punch were placed in RPMI-1640 medium supplemented with 10% heat-inactivated fetal bovine serum, 2 mM of L-glutamine, 10 mM of MEM non-essential amino acids, and 100 U/mL of penicillin/streptomycin (all from Gibco, ThermoFisher Scientific, Waltham, MA, USA). Samples were subsequently minced with a scalpel (~1 mm fragments) and maintained overnight at 37 °C in a humidified incubator at 5% CO_2_ in the presence of collagenase P (cat. 11213865001, Roche, Mannheim, DE, Germany) to a final concentration of 1 μg/mL. Cells were then resuspended in RPMI-1640 full medium, supplemented with 1 μg/mL of ionomycin (cat. 407952, Merck, Darmstadt, DE, Germany), 10 ng/mL of phorbol-12-myristate-13-acetate/PMA (cat. P8139, Merck, Darmstadt, DE, Germany), 5 μg/mL of brefeldin A (cat. 203729, Merck, Darmstadt, DE, Germany), and 5 μM of monensin (cat. M5273, Merck, Darmstadt, DE, Germany), and incubated for 4 h at 37 °C to stimulate cytokine production. Stimulation was halted with ice-cold 1 mM EDTA and 1 mM CaCl_2_ in phosphate-buffered saline (PBS), pH 7.4. Cells were then dispersed by gentle pipetting, followed by centrifugation at 300× *g* for 10 min at room temperature, and resuspended in 1 mL of bovine serum albumin (BSA) 1% (*w*/*v*) in PBS, preceding subsequent staining in preparation for analysis by flow cytometry.

### 2.2. Flow Cytometry

To define the T cell subsets within the studied samples (BCCs, SCCs, and normal skin), a sequential gating strategy was employed. The first gate was applied on the combined forward scatter (FSC) plus side scatter (SSC) plots. A forward scatter area (FSC-A) vs. side scatter area (SSC-A) plot was applied to identify lymphocytes (TILs; typically, 30–35% of the whole cell population) by size and granularity. Subsequently, a gate on the FSC-A vs. forward scatter height (FSC-H) plot was used to identify single cells (typically 99%). Next, live/dead discrimination was performed using staining for viability with the LIVE/DEAD Fixable Dead Cell Stain Kit (Invitrogen, Waltham, MA, USA), according to the manufacturer’s instructions. Events positive for the dead cell stain were excluded, ensuring analysis of viable cells only (90% of remaining events).

Following viability selection, a gate was applied to the CD3 channel to identify T cells (65–80% of live cells). Within the CD3+ population, further gating was performed to identify memory cells, based on the expression of CD45RO, and to identify γδ cells, based on the expression of TCR (T cell receptor) γδ. Additionally, within the CD3+ population, specific T cell subsets were identified based on expression of the CD4 and CD8 markers. The identification of CD4+ T cell subtypes was further based on the expression of IFN-γ (Th1 cells), IL-4 (Th2 cells), IL-17 (Th17 cells), IL-9 (Th9 cells), and FoxP3 (Treg cells). A minimum of 10,000 events was analyzed per experiment.

For surface staining, non-specific Fc-mediated interactions were blocked with human Fc receptor-binding inhibitor (cat. 14-9161-73, eBioscience, San Diego, CA, USA), and cells were incubated with directly conjugated primary antibodies directed against specific surface T cell markers (detailed below). For intracellular staining, cells were exposed to Foxp3/Transcription Factor Fixation/Permeabilization Concentrate and Diluent Kit (cat. 00-5521-00, eBioscience, San Diego, CA, USA) before incubation with directly conjugated primary antibodies (detailed below).

Thus, to identify infiltrating T cell subpopulations, the standard combination of fluorescent probes comprised LIVE/DEAD Fixable Near-IR plus the following fluorochrome-coupled monoclonal antibodies: Brilliant Violet 785 anti-human CD3, Brilliant Violet 711 anti-human CD8a, PE anti-human TCR γδ, Brilliant Violet 510 anti-human CD45RO, Brilliant Violet 605 anti-human CD4, Brilliant Violet 65 anti-human IFN-γ, Alexa Fluor 488 anti-human IL-4, and Alexa Fluor 647 anti-human IL-9 (all from Biolegend, San Diego, CA, USA), as well as eFluor 450 anti-human IL-17A and PerCP-Cyanine5.5 anti-human Foxp3 (both from eBioscience, San Diego, CA, USA).

Compensation was performed using antibody capture beads (Rainbow Calibration Particles—8 beads, Biolegend, San Diego, CA, USA). Acquisition was performed using a Fortessa X20 cytometer and BD FACS Diva Software v8.0. Data analyses were carried out using FlowJo v10.

### 2.3. Immunohistochemistry (IHC)

BCCs (n = 12) and SCCs (n = 10) were randomly selected from the entire sample collection and analyzed by flow cytometry, and NS samples (n = 10) were routinely fixed in 4% neutral buffered formaldehyde. For immunohistochemistry analyses, 3 μm paraffin-embedded sections were routinely processed for staining with antibodies specific for either CD3, CD4, CD8, or FoxP3, as further detailed.

Paraffin sections were mounted onto Superfrost Plus slides (Ref. J1800AMNZ, Thermo Fisher Scientific, Waltham, MA, USA) and baked at 65 °C for 60 min to enhance adherence prior to staining by immunohistochemistry. Immunostaining was performed in the BenchMark ULTRA platform (Ventana Medical Systems, Roche Tissue Diagnostics, Tucson, AZ, USA). Antibodies used in this study were rabbit polyclonal anti-human CD3 (cat. A045229-2, Agilent Technologies Singapore, Singapore), rabbit monoclonal anti-human CD4 (clone SP35, Ventana Medical Systems, Roche Tissue Diagnostics, Tucson, AZ, USA), mouse monoclonal anti-human CD8 (clone 4B11, cat. PA0183, Leica Biosystems, Newcastle, UK), and rabbit monoclonal anti-human FoxP3 (clone SP97, Life Technologies Europe BV, Bleiswijk, Netherlands). The OptiView DAB IHC Detection Kit (Ventana Medical Systems, Roche Tissue Diagnostics, Tucson, AZ, USA), a biotin-free system, was used for detecting primary antibodies.

To assess the density of cells staining for CD3, CD4, CD8, and FoxP3 in NMSC and normal skin in tissue sections, 10–15 images were obtained per sample at 400× magnification. Each image was thus representative of an area of 0.33 × 0.25 mm. To overcome the chance of assessing overlapping areas, one 400× magnification field was left between each two sequentially collected images. In NMSCs, we ensured that 8–10 images per sample included the stroma surrounding tumor cell nests, to quantify stromal TILs (*St* area), and that 3–5 images included mostly cancer cell nests, to assess intra-tumoral TILs (*It* area). In normal skin controls, images were sequentially obtained from the dermo-epidermal junction and upper dermis. Areas of ulceration were excluded from the analysis.

Images were obtained with a Leica ICC50 HD bright-field digital microscope equipped with the Leica LAS EZ software v3.0. The percentage of cells expressing the selected markers (CD3, CD4, CD8, and FoxP3) was scored semi-quantitatively at 400× magnification field, as follows: 0 (<1%), 1 (1–5%), 2 (6–30%), or 3 (>30%). The assessment was performed independently by two investigators (D.C. and D.S.). To aid in the semi-quantitative evaluation of stained areas, a guide grid was placed adjacent to, or overlapping, the microscope field of view.

### 2.4. Statistical Analyses

After data collection and data management, statistical analyses were performed using the one-way ANOVA test (normally distributed data) or the Kruskal–Wallis non-parametric test (non-normally distributed data) in IBM-SPSS Statistics, v.26. A Type I (α) error probability of 0.05 was used. When the one-way ANOVA test was statistically significant, multiple comparisons were performed with Scheffe’s or Games–Howell’s post-hoc tests. When the Kruskal–Wallis test was statistically significant, multiple comparisons were performed with the Mann–Whitney U test, and a Bonferroni correction was used, as follows: 0.05/3 = 0.017 for tumor types, and 0.05/4 = 0.0125 for tumor subtypes.

## 3. Results

### 3.1. Patients and Clinical Samples

A total of 181 samples were collected (BCCs, n = 118; SCCs, n = 33; NS, n = 30). The study population included 94 (52%) men and 87 (48%) women, with a mean age of 73.5 years in the BCC group, 84.6 years in the SCC group, and 43.3 years in the NS group (Table 1). BCCs and SCCs were chiefly located on the head and neck areas (>80%; Table 1).

### 3.2. BCC and SCC Share a Similar Inflammatory Immunophenotype

There is supportive evidence for the spectrum of TILs within the tumoral microenvironment influencing the progression of disease and response to immunotherapies [17,43,73,74]. In NMSCs, namely BCCs and SCCs, the characterization of TIL subpopulations is still fragmentary, and a global overview is critically missing. Based on past evidence obtained on NMSCs and other cutaneous and non-cutaneous tumors, we anticipated a scenario in which subsets of TILs endowed with either pro-tumoral or anti-tumoral roles coexisted, likely in imbalanced proportions relative to the normal tissue of origin [2,36,75].

**Figure 1:**
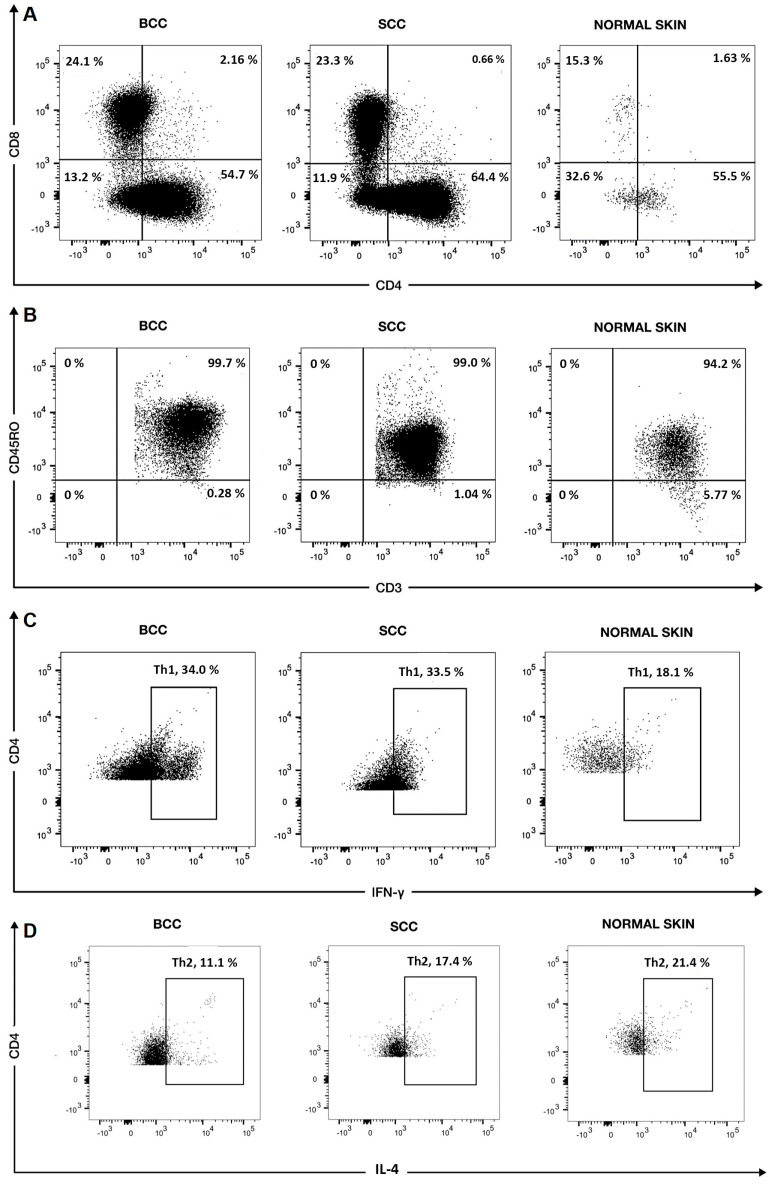

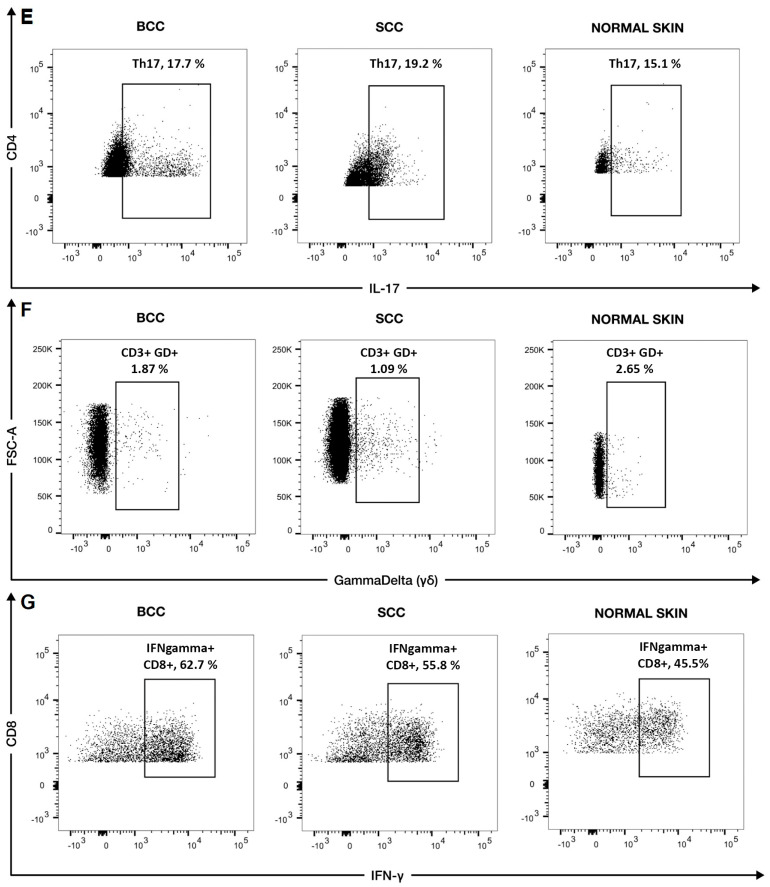
Flow cytometry charts from one representative BCC, one SCC, and one NS sample. In the dot plots, percentages denote the frequency of events/cells within selected regions relative to the total depicted events. Shown are percentages of (**A**) CD4+ and CD8+ cells within CD3+ cells, (**B**) CD45RO+ cells within the CD3+ population, (**C**) IFN-γ+ cells within the CD4+ population (Th1 cells), (**D**) IL-4+ cells within the CD4+ population (Th2 cells), and (**E**) IL-17+ cells within the CD4+ population (Th17 cells). In (**F**), the graphs depict the combined forward scatter plot (FSC-A) of lymphocytes, featuring the expected dispersion of cell sizes (y axis) and concurrent staining for the TCR γδ (GD). The lymphocyte population was gated from a prior compound scatter plot (forward plus side scattering). Also shown are the percentages of IFN-γ+ cells within the CD8+ population (**G**). In the graphs, y and x axes depict the logarithm of relative fluorescence intensities.

**Figure 2:**
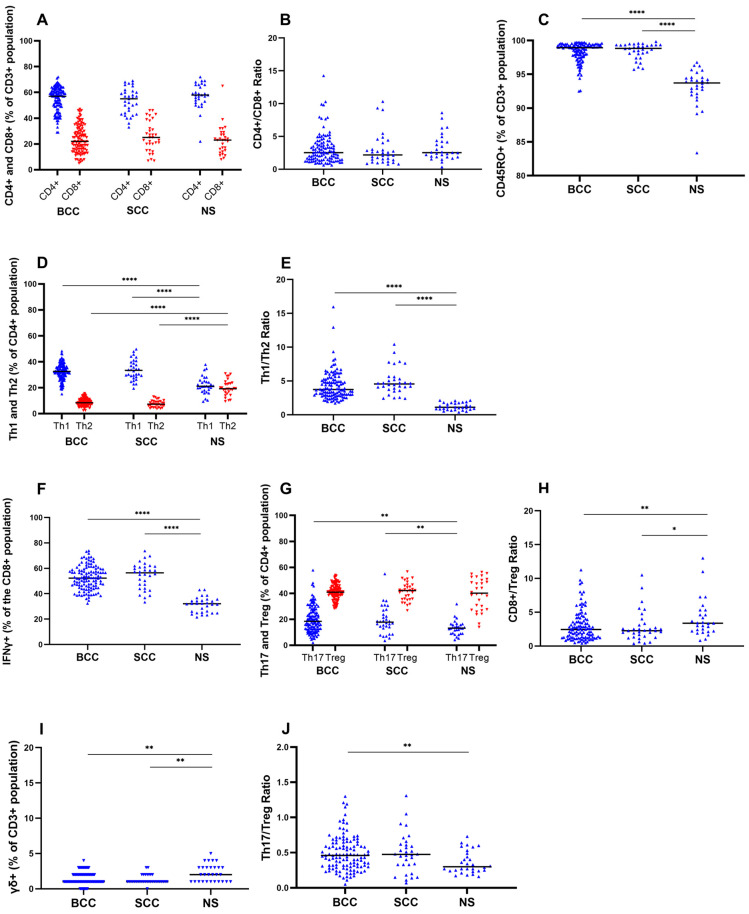
NMSCs feature altered percentages of CD4 and CD8 T cell subpopulations relative to NS controls. (**A**–**J**) Graphs displaying frequencies and ratios of T cell subsets in BCCs, SCCs, and NS. (**A**) Percentages of CD4+ and CD8+ cells after gating for CD3+ T cells. (**B**) Ratios between CD4+ and CD8+ T cells. (**C**) Percentage of CD45RO+ cells after gating for CD3+ T cells. (**D**) Percentages of Th1 and Th2 cells after gating for CD4+ T cells. (**E**) Ratios between Th1 and Th2 T cells. (**F**) Percentages of IFNγ+ T cells after gating for CD8+ T cells. (**G**) Percentages of Th17 and FoxP3+ Treg cells after gating for CD4+ T cells. (**H**) Ratios between CD8+ T cells and Treg cells. (**I**) Percentages of T cells expressing the γδ receptor after gating for CD3+ T cells. (**J**) Ratios between Th17 and Treg cells. Horizontal bars represent medians, * *p* < 0.05, ** *p* < 0.01, **** *p* < 0.0001.

Herein, we used flow cytometry (Figure 1) to address whether the proportions of CD8+ and CD4+ T cells, major subtypes of CD3+ T cells, varied between NMSCs and NS. Remarkably, the percentages of CD4+ and CD8+ cells within the CD3+ T cell population were similar among BCCs, SCCs, and NS, with clearly higher frequencies of CD4+ relative to CD8+ T cells (Table 2; Figure 2A). Accordingly, the CD4/CD8 ratio was not significantly different between BCCs (median: 2.5; range: 0.6–14.3), SCCs (median: 2.2; range: 0.8–10.3), and control NS (median: 2.5; range: 0.3–8.6; Table 2; Figure 2B).

As expected, CD3+ T cells expressing the memory marker CD45RO featured a slight but significant increase in NMSCs compared to NS (Table 2; Figure 2C).

Considering the relatively constant proportion of CD4+ T cells within the T cell (CD3+) infiltrates in both NMSCs and NS, we asked whether the frequencies of CD4+ T cells that were Th1 and Th2 cells, and the Th1/Th2 ratio, of major relevance in tumor immunobiology were also maintained. We found that both BCCs and SCCs differed from NS by harboring a T cell infiltrate significantly enriched for Th1 cells (IFN-γ+ CD4+ T cells; BCCs, median: 32.6%, range: 15.1–48.2; SCCs, median: 33.4%, range: 19.4–49.7; NS, median: 21.2%, range: 9.3–37.8; Table 3; Figure 2D). Inversely, a lower proportion of Th2 (IL-4+ CD4+ T cells) cells was detected in both tumor types compared to NS (BCCs, median: 8.3%, range: 2.4–15.6; SCCs, median: 7.3%, range: 3.7–13.1; NS, median: 19.2%, range: 9.4–30.9; Table 3; Figure 2D). Overall, both BCCs and SCCs featured a significantly higher Th1/Th2 ratio (~four-fold) than NS (Table 3; Figure 2E). Of note, this higher ratio was maintained in BCCs and SCCs, irrespective of their histologic subtype (Tables S2 and S4).

We subsequently asked whether CD8+ T lymphocytes, mostly connoted with cytotoxic functions, that could produce the prototypical cytokine IFN-γ under stimulation conditions (see the Materials and Methods section) were reduced in NMSCs relative to NS. We thus compared, in NMSCs and control NS, the percentages of cells that were CD8+ and IFN-γ+ within the global CD3+ T cell population. Surprisingly, we found a significant enrichment in IFN-γ+ CD8+ T cells in both BCCs (mean: 52.3%; range: 32.4–73.9) and SCCs (mean: 56.5%; range: 33.4–73.7) in comparison with NS (mean: 32.1%; range: 21.5–43.1; Table 2). We note that, considering the overall similar percentages of T cells that were CD8+ between NMSCs and control NS (Table 2; Figure 2A), these data highlight the presence of a significant fraction of CD8+ T cells prone to IFN-γ production within NMSCs (Figure 2F). Importantly, the enrichment of IFN-γ+ CD8+ T cells was observed across all subtypes of BCCs (superficial, nodular, micronodular/infiltrative, and other subtypes; Table S1) and SCCs (in situ, well differentiated, moderately/poorly differentiated, and other subtypes; Table S3) analyzed herein.

We next tested whether Tregs, another subpopulation of CD4+ T cells, and the ratio between CD8+ T cells and Tregs (CD8/Treg ratio), whose decrease is held as indicative of a pro-tumoral environment [45,46,47,76,77], were also altered in NMSCs.

While Tregs contributed the highest percentage of cells among CD4+ T cells, their proportions within the CD4+ population did not vary significantly between BCCs (median: 40.9%; range: 28.3–54.0), SCCs (median: 42.1%; range: 26.8–56.7), and control NS (median: 40.2%; range: 14–56.3; Table 3; Figure 2G). This trend was consistent among the major histologic subtypes of BCCs and SCCs (Tables S2 and S4). Despite this, the CD8/Treg ratio was significantly lower in BCCs and SCCs compared to NS (Table 3; Figure 2H), thus providing an immunosuppressive component to the TME in NMSCs.

Subsequently, we checked whether γδ T cells were also altered in BCCs and SCCs relative to NS. Indeed, a significant depletion of γδ T cells within the global CD3+ T cell population was observed in BCCs (median: 1%; range: 0.0–4.0) and SCCs (median: 1%; range: 0.0–3.0) compared to NS (median: 2%; range: 1.0–5.0; Table 2; Figure 2I). Considering the overall low frequency of γδ T cells (1 to 2% of CD3+ T cells), we were unable to further consistently characterize this subpopulation with respect to cytokine production.

Since the NMSC samples analyzed herein were, as expected, mainly localized in the head and neck, and the control NS samples were collected from the trunk and limbs, we also compared the frequencies of T cell subpopulations between NS and NMSCs originating from the trunk and limbs. Although necessarily restricted to a smaller number of NMSC samples, this analysis showed percentages of specific T cell subpopulations similar to the whole set of NMSCs (Tables S5 and S6).

In sum, when compared to normal skin, BCCs and SCCs featured a similar percentage of CD8+ and CD4+T cells (and similar CD4+/CD8+ ratios) but an imbalanced profile of CD4+T cell subpopulations, namely, a prominence of Th1 cells over Th2 cells. A significant enrichment of IFN-γ-producing CD8+ T cells was found in both BCCs and SCCs, in parallel with a lower CD8/Treg ratio and a depletion of γδ T cells in the tumoral microenvironment.

### 3.3. Th17 in the NMSC Tumor Microenvironment

Although both Th17 and Th9 subsets of T cells have been implicated in tumorigenesis, the extent of their involvement in NMSC has remained poorly characterized [61,62,63]. In this research, the expression of IL-9 by T cells in BCCs and SCCs was barely identified by flow cytometry. On the contrary, a subset of CD4+ T cells expressing IL-17 (Th17 cells) was found in both NMSCs and NS. Importantly, the percentage of Th17 cells was significantly higher in BCCs and SCCs than in NS (BCCs, median: 18.5%, range: 1.9–57.9; SCCs, median: 17.8%, range: 3.5–55.0; NS, median: 13.2%, range: 4.1–31.8; Table 3; Figure 2G). A more detailed analysis showed that this enrichment of Th17 cells was significant for most histological subtypes of BCCs (e.g., superficial and micronodular/infiltrative) and for the most undifferentiated subtypes of SCCs (Tables S2 and S4). When comparing the relevant Th17/Treg ratio in BCCs and SCCs against NS, this was significantly higher only in BCCs (Table 3; Figure 2J).

### 3.4. T Cells Infiltrate the Periphery of BCCs and SCCs

While flow cytometry provides valuable data on relative proportions of tissue-infiltrating T cell subsets, information regarding their densities (i.e., numbers of specific T cell subsets per area) in different samples is mostly missed. Also missed are the spatial relationships between host-derived TILs and cancer cells.

Herein, we complemented our flow cytometry analyses with IHC to separately quantify inflammatory cells staining for CD3+, CD4+, CD8+, or Foxp3+ (Tregs) using a semi-quantitative scoring index (from 0 to 3) that reflects cell densities (see the Materials and Methods section).

**Figure 3:**
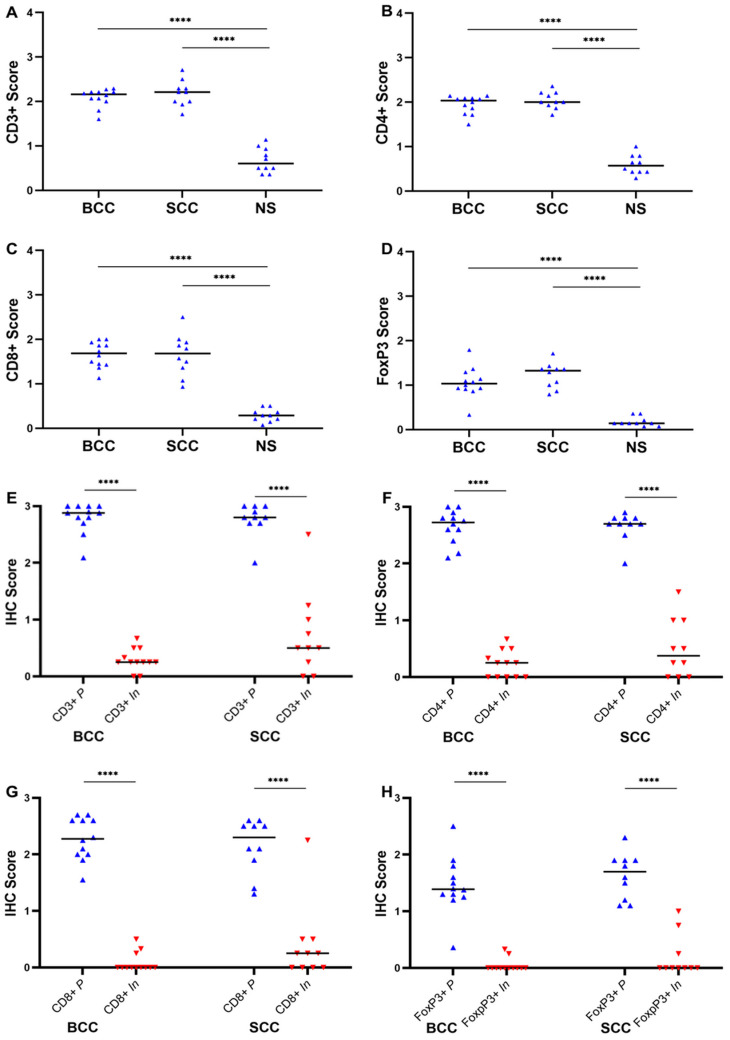
Infiltrates of CD3+, CD4+, CD8+, and FoxP3+ T cells are denser in BCCs and SCCs than in NS and are mainly localized at the tumor periphery. (**A**–**D**) Graphs showing IHC scores in BCCs and SCCs compared to NS for (**A**) CD3+, (**B**) CD4+, (**C**) CD8+, and (**D**) FoxP3+ T cells. (**E**–**H**) Graphs representing the spatial distribution of the infiltrates of (**E**) CD3+, (**F**) CD4+, (**G**) CD8+, and (**H**) FoxP3+ T cells at the inner areas of the tumor (*In*) and at the tumor periphery (*P*). Horizontal bars represent medians, **** *p* < 0.0001.

**Figure 4:**
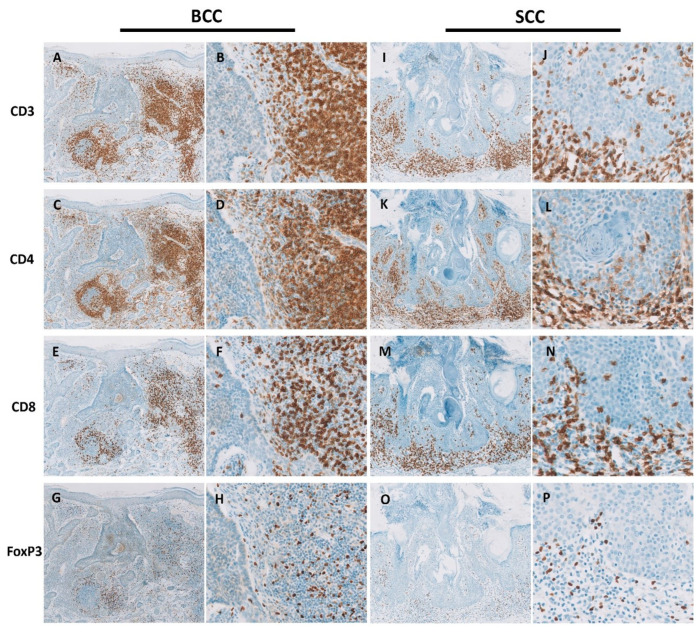
BCCs and SCCs feature a dense stromal T cell infiltrate. Shown are contiguous sections of exemplary samples of BCC stained for CD3 ((**A**), 50×; (**B**), 200×), CD4 ((**C**), 50×; (**D**), 200×), CD8 ((**E**), 50×; (**F**), 200×), and FoxP3 ((**G**), 50×; (**H**), 200×), and of SCC, also stained for CD3 ((**I**), 50×; (**J**), 200×), CD4 ((**K**), 50×; (**L**), 200×), CD8 ((**M**), 50×; (**N**), 200×), and FoxP3 ((**O**), 50×; (**P**), 200×). Note the overall paucity of T cells within the tumoral epithelia. Magnifications provided correspond to original magnifications.

**Figure 5:**
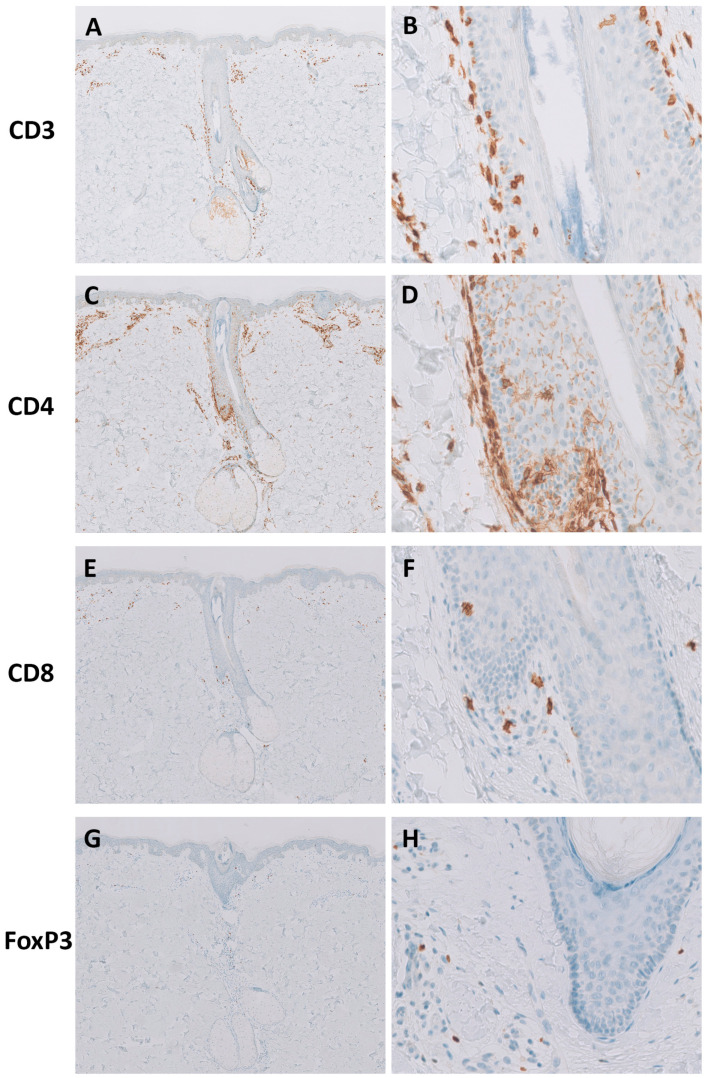
T cell populations in normal skin. Shown are contiguous sections of normal skin samples stained for CD3 ((**A**), 50×; (**B**), 200×), CD4 ((**C**), 50×; (**D**), 200×), CD8 ((**E**), 50×; (**F**), 200×), and FoxP3 ((**G**), 50×; (**H**), 200×). Lymphoid infiltrates are scarce and mainly circumscribed to the perivascular and perifollicular areas. Magnifications provided correspond to original magnifications.

The median scores for infiltrating CD3+ cells both in BCCs (median: 2.2, range: 1.6–2.3, *p* < 0.0001) and SCCs (median: 2.2, range: 1.7–2.7, *p* < 0.0001) significantly outweighed the score for this population in NS (median: 0.6, range: 0.4–1.1; Table 4; Figure 3A). Whereas BCCs and SCCs had similar scores for infiltrating CD4+ cells (BCC median score: 2.0, range: 1.5–2.1; SCC median score: 2.0, range: 1.7–2.4), their median scores were significantly higher than in NS (median: 0.6; range: 0.3–1.0; Table 4, Figure 3B). Similarly, both CD8+- and FoxP3+-infiltrating T cells had, respectively, significantly higher median scores in BCCs (CD8+, median: 1.7, range: 1.1–2.0; FoxP3+ median: 1.0, range: 0.3–1.8) and SCCs (CD8+, median: 1.7, range: 0.9–2.5; FoxP3+, median: 1.3, range: 0.8–1.7) than in NS (CD8+, median: 0.3, range: 0.1–0.5; FoxP3+, median: 0.1, range: 0.1–0.4; Table 4; Figure 3C,D). Thus, the median scores for CD4+, CD8+, and Treg cells were significantly higher in BCCs and SCCs than in NS, while they were identical between the two tumor types (Table 4). These data are consistent with an overall denser T cell infiltrate in NMSCs (Figure 4) relative to NS (Figure 5).

We subsequently analyzed the scores for CD3+, CD4+, CD8+, and FoxP3+ cells localizing either at the inner area of the tumor (*In* area) or at the tumor periphery (*P* area) (see the Materials and Methods section for details). In both SCCs and BCCs, the median scores for these populations were consistently higher for the *P* area than for the *In* area (Table 4; Figure 3E–H and Figure 4). BCCs and SCCs featured identical scores when compared at the *In* area and the *P* area (Table 4; Figure 3E–H).

Altogether, these data showed that BCCs and SCCs share a dense T cell infiltrate of similar composition regarding broad T cell subsets, namely, CD8+, CD4+, and FoxP3+, essentially confined to the tumor periphery.

## 4. Discussion

Herein, we have provided evidence that BCCs and SCCs share similar T cell infiltrates, which in turn differed from those observed in NS. Using the NS comparator, several findings stood out in NMSCs relating to both cell densities and relative frequencies of T cell subsets, namely: (1) an overall denser T cell infiltrate, comprising CD4+ and CD8+ T cells, and FoxP3+ Tregs, (2) an imbalanced profile of CD4+ T cell subsets, which included a predominance of Th1 over Th2 helper-type T cells, and (3) a higher frequency of Th17 cells. Compared to NS controls, NMSCs also featured, (4) a significant enrichment of IFN-γ-producing CD8+T cells, in parallel with (5) a lower CD8/Treg ratio and (6) a depletion of γδ T cells.

In the current study, and as noted previously [2,36,75], both significant pro- and anti-tumor subsets of TILs were found to coexist in the TME of NMSCs, as discussed below.

As is typical for cancers caused by exogenous factors (e.g., UV light), both BCCs and SCCs displayed a high mutation burden, and thus an expected increase of neoantigens and the role of the immune system in tumor control [78]. Accordingly, under immunosuppression regimens, the incidence of NMSCs was shown to rise dramatically, in particular for SCCs, which may reach 65- to 250-fold compared with the general population [78,79,80,81]. Moreover, despite supportive evidence for immunoediting in selection of cancer cells that evade the host immune system [11,12], the recent success of immune-based therapies, namely in NMSCs, strongly argues against development of full tolerance in immunocompetent hosts [11,12,67,68,69,82].

Considering the relevance of TILs in cancer development and progression, several studies have previously addressed the characterization of TILs in the tumor microenvironment (TME) of NMSCs. We note, however, that past studies on the subject have yielded controversial results, namely, on the predominance of either CD8+ or CD4+ T cells and, among the CD4+ T cell population, whether the immune response was polarized toward Th1- or Th2-type helper T cells [21,32,33,40,61,83]. Additionally, whether the frequency of Tregs within the CD4+ T cell population was higher in NMSCs than in NS has remained debatable [2,36,48]. This may have stemmed from several factors, including different experimental approaches for T cell analysis, the focus on either BCCs or SCCs, and the use of different control tissues for comparison between T cell subsets (e.g., skin vs. blood), among others.

Likewise, the tumor microenvironment of BCCs has been found as either enriched or depleted in CD8+ T cells [21,61]. In another analysis, a T cell infiltrate enriched for CD8+ T cells was found only in SCCs, not in BCCs [20]. CD4+ T cells were described as increased in BCCs [61,83] and in SCCs [61] in a small number of tested samples. However, to what extent these CD4 T cells corresponded to main T helper subtypes, namely, Th1 and Th2 T cells, has not been directly addressed.

Herein, we studied a large number of NMSC samples, and used a combined approach of flow cytometry, aimed at providing frequencies (percentages) of several T cell subpopulations, and immunohistochemistry to better assess densities of major T cell subsets. Additionally, we used normal skin as the comparator. We found that the percentages of CD3+ T cells that were either CD4+ or CD8+ did not vary significantly between BCCs, SCCs, and NS, with CD4+ T cells consistently occurring at higher frequencies than CD8+ T cells in all groups (Table 2). Accordingly, CD4/CD8 ratios were maintained between BCCs (median, 2.5; range, 0.6–14.3), SCCs (median, 2.2; range, 0.8–10.3), and healthy skin (median, 2.5; range, 0.3–8.6; Table 2). Of note, in NMSCs, this CD4/CD8 ratio was maintained despite increased densities of both CD4+ and CD8+ T cells relative to normal skin, as assessed by immunohistochemistry (Table 4). The difference between CD4+ and CD8+ frequencies within NMSCs and NS was less obvious using immunohistochemistry since this approach was based on a semi-quantitative scoring system lacking quantitative power for densities above 30%.

Importantly, this balanced increase of CD4 and CD8 T cells in the microenvironment of NMSCs was reached, in the case of CD4 T cells, at the expense of an increased frequency of Th1 relative to Th2 cells. Indeed, although the Th1/Th2 ratio in normal skin slightly favors Th1 cells (mean,1.1; range, 0.4–2.1), in both BCCs and SCCs, an increment of ~four-fold in the Th1/Th2 ratio was observed relative to normal skin, irrespective of the histologic subtype (Table 3).

The increased frequency of Th1 cells in NMSCs disclosed by our data supports the maintenance of a relevant anti-tumoral activity within the TME. There is current agreement on Th1 cells being committed to anti-tumor activities due to their roles in production of IFN-γ, macrophage activation, and priming and activation of cytotoxic CD8+ T cells, and the generation and maintenance of long-lived CD8+ T cells [20,84,85]. Accordingly, increased expression of IFN-γ has been reported in regressing BCCs [32,33], although besides Th1 cells, the production of IFN-γ is shared with other immune cell types, namely, activated CD8 T cells, natural killer T cells, and macrophages [86,87]. Our findings, supporting a shift from a Th2 to a Th1 dominance in NMSCs, may disclose potential targets for developing new immune-based treatments for these tumors.

As mentioned above, we observed a predominance of the frequency of CD4+ T cells over CD8+ T cells in both BCCs and SCCs, as well as in NS (Table 2). Although CD8+ T cells are mostly connoted with cytotoxic and tumoricidal activities, namely, in NMSCs [2,36,88], a wealth of past research has disclosed a major role also for CD4+ T cells in tumor immune surveillance with relevance to prognosis. While a rich T cell (CD3+) infiltrate correlates, per se, positively with a longer survival in several cancers, concurrent enrichment in CD8+ plus CD4+ T cells seems to be required for a better prognosis in some cancers, including head and neck squamous cell carcinomas (HNSCCs) [89]. Finally, in a mouse model, PD-1 blockade was shown to not only increase CD8+ but also CD4+ T cells during regression of SCCs [88].

Altogether, the above-mentioned findings support the occurrence of a relevant anti-tumoral T cell component within the TME of NMSCs.

Our data, obtained from a large number of NMSC samples, showed that although Tregs comprised a substantial fraction of the CD4+ T cell population, within this CD4+ population, the percentage of Tregs was not significantly different between BCCs, SCCs, and control NS (Table 3). This constancy was still observed when BCCs and SCCs were stratified according to histological subtype (Tables S2 and S4).

The issue of whether the percentage of Tregs within the T cell compartment varies between NMSCs and NS has been a matter of debate [36]. We note that in a recent study, NMSCs were also found not to have higher frequencies of Tregs than NS, in line with our data [40]. However, the density of Tregs, which is best judged using immunohistochemistry, was significantly higher in BCCs and SCCs than in control skin samples, mirroring the parallel increase of CD4+ T cell densities in tumors (Table 4). Similar results were previously reported using immunohistochemistry on nodular BCCs [21,48] and on SCCs [22]. As observed for the total CD4+ T cell population, and for CD8+ T cells, most Foxp3+ Tregs localized to the tumoral stroma (Figure 3E–H; Table 4).

Regulatory CD4+ T cells are recognized as acting mostly as suppressors of anti-tumor responses, at least partially through secretion of TGF-b and IL-10 [43,90], and are deemed as major contributors to the mostly immunosuppressive cytokine milieu described for NMSCs [2,21,36,48,50]. Additionally, consistent with the suppressive function of Tregs in SCCs, treatment with the Toll-like receptor (TLR)7 agonist, imiquimod, led to a decrease in frequency (and function) of Tregs, concurrent with an increment in CD8+ T cells [22]. An enrichment for Tregs in the tumor microenvironment has thus typically been associated with a worse prognosis in several types of cancer, including murine and human SCCs [2,36,50,73,75,91]. We further observed a decreased CD8/Treg ratio in BCCs and SCCs (Table 3), an imbalance typically connoted with pro-tumor immunity [76].

With respect to Th17 T cells as a component of the microenvironment of NMSCs, infiltrating IL-17+ T cells have been previously identified in the analysis of a small number of samples comprising both BCCs and SCCs [61]. However, the identity of the IL-17+ cells as Th17 could not be ascertained since co-staining for CD4 was not assessed [61]. Eventually more incisive in this regard was the demonstration by immunohistochemistry of an enrichment of Th17 (CD4+ Th17+) cells in the stroma of BCCs relative to normal skin. SCCs were not, however, included in the analysis [33].

In the present research, using flow cytometry after stimulation of interleukin production, we found that within the CD4+ T cell population, the frequencies of Th17 cells were significantly higher in BCCs and SCCs relative to control skin (Table 3); of note, this difference was sharper for BCCs (BCCs vs. NS, *p* = 0.001) than for SCCs (SCCs vs. NS, *p* = 0.007).

Additionally, when NMSCs were analyzed by histological subtype, the relative enrichment in Th17 cells was observed only in the least differentiated subtypes of SCCs but was sustained in most subtypes of BCCs (Tables S2 and S4).

Interestingly, in mouse models of carcinogen-induced skin cancer, IL-17 was shown to promote both the development and growth of tumors, and the production of pro-tumoral cytokines and proliferation of cancer cells [62,63]. These experimental data lend support for a pro-tumoral role of IL-17 in NMSCs. Together with our data gleaned from the analysis of clinical samples, this raises the relevant possibility of IL-17 becoming a target for immunotherapy, eventually as part of combination schemes in advanced cases of NMSCs.

Due to their cytotoxic action and ability to produce IFN-γ, γδ T cells are considered relevant players in anti-tumor immunity [51,55] and their depletion in tumors expectedly promotes tumor progression. However, γδ T cells may also polarize toward pro-tumor phenotypes depending on the cytokine milieu [59,92,93,94]. Considering the scarcity of γδ T cells in our samples, we could not reliably discriminate between distinct phenotypes based on their signature markers (e.g., IL-10, IL-17, and IFN-γ).

Using the canonical memory T cell marker CD45RO, we found that the vast majority of CD4+ and CD8+ T cells (>98%) infiltrating NMSCs featured a memory phenotype (Table 2). The difference relative to control NS was slight, yet significant (*p* < 0.001). This is not unexpected considering the long period of growth of these tumors, and thus of chronic exposure of the host immune cells to cancer cells and their cognate neoantigens. Similar observations have been recently reported in cutaneous SCCs, where naive T cells were found at negligible frequencies in both SCCs and NS [95]. It was further highlighted that the majority of CD8+ and CD4+ T cells infiltrating SCCs (and NS) correspond to tissue-resident memory T (Trm) cells featuring an effector phenotype [95,96,97,98]. Additionally, in non-cutaneous solid tumors and in human melanoma, a large fraction of CD8+ TILs were comprised of exhausted Trm cells (CD103+CD39+) that had effector phenotypes and possessed anti-tumor reactivity [99,100]. Although in some non-cutaneous solid tumors, infiltration by this subset of CD8+ TILs correlated with a better prognosis, the reverse was observed for cutaneous SCCs [95].

Possibly reflecting the absence of cases of advanced disease in our series, we found that within the global (CD3+) T cell population, CD8+ T cells producing IFN-γ occurred at significantly higher frequencies in SCCs (mean, 56.5%; range, 33.4–73.7) compared to control NS (mean, 32.2%; range, 21.5–43.1), and the same was true for BCCs (mean, 52.3%; range, 32.4–73.9; Table 2). These data, although not excluding coexistence of dysfunctional states (e.g., exhaustion), strongly support the presence within the TME of NMSCs of substantial populations of CD8+ T cells capable of producing IFN-γ, a major anti-tumoral cytokine. We suggest that these cells correspond to the CD8+ cytotoxic T cells identified in other solid tumors, including HNSSCs, and shown to secrete IFN-γ while recognizing tumor-specific antigens and exerting efficacious cytotoxic activity against autologous cancer cells [99,100].

Our analysis by immunohistochemistry disclosed a relevant immune host response in NMSCs predominantly confined to the periphery of tumors (Table 4). These features of NMSCs fit into the concept of immune-excluded tumors, in which T cells barely penetrate the tumor parenchyma [101]. Of note, immune-excluded tumors are currently deemed as irresponsive to immune therapies [101]. Thus, the distribution of TILs in NMSCs shown herein would anticipate a poor response to treatment with immune checkpoint inhibitors, e.g., anti-PD1. However, these drugs have been successfully used in the treatment of advanced SCCs [67,82] and BCCs [68,69], strongly arguing in favor of T cells playing a major role in the TME of NMSC.

It was recently reported that anti-PD-1 immunotherapy of advanced SCCs and BCCs promoted recruitment of novel anti-tumoral T cell clonotypes into the TME, and that at least a fraction of these clones was already present in circulating blood [102]. Central memory T (Tcm) cells seem well placed to contribute to incoming clonotypes considering their capacity to recirculate between secondary lymphoid organs, blood, and peripheral tissues, namely, the skin, where they have been shown to exert immune-surveillance and effector functions [26,84,96,97,98,103,104].

In the present study, we did not address the reactivity of NMSC-infiltrating TILs against tumor-specific antigens, and this may pose a limitation of our research. Another limitation to this study is the incomplete body region matching between control and tumor samples. Notwithstanding this, we are confident that our analysis will provide a useful roadmap for future research on TILs associated with clinically non-advanced NMSCs.

Future work will help to clarify how immunotherapy affects (1) the balance between bystander T cells, presumed to comprise a diverse array of clonotypes within the TME of NMSCs [105], tumor-specific resident clonotypes of exhausted CD8 T cells, and incoming new clonotypes of CD8 T cells, and (2) the dynamics between intra-tumoral and stromal TILs.

## 5. Conclusions

In summary, the similarities between the T cell infiltrates in BCCs and SCCs support the idea that these tumors induce a similar T-cell-mediated host response. Both BCCs and SCCs featured a denser infiltrate of T cells, including Foxp3+ Tregs, than control normal skin. Frequencies of CD4+ T cells were significantly higher than those of CD8+ T cells in both types of NMSCs, similar to normal skin. However, in contrast to normal skin, within the CD4+ T cell population, the percentages of IFN-γ+ Th1 cells were significantly higher (~four-fold) than those of Th2 cells in BCCs and SCCs. NMSCs also featured higher frequencies of IFN-γ+ cells that were CD8+ T cells than normal skin. Additionally, relative to normal skin, most subtypes of BCCs and the least differentiated SCCs showed a significantly higher fraction of Th17 cells within the CD4+ T cell population. Altogether, these data show that despite the presence of pro-tumoral features within the tumor microenvironment of NMSCs, namely, a dense T cell infiltrate of Tregs and a reduced frequency of γδ T cells, this may be counterbalanced by an enrichment in IFN-γ-producing Th1 and CD8+ T cells. These latter cell types have the potential to lead the IFN-γ-driven anti-tumoral response observed in NMSCs under immunotherapy.

Our results pave the way for future research on a potential novel therapeutic strategy combining immune checkpoint inhibition and IL-17 pathway targeting. This strategy would mostly benefit advanced and treatment-refractory NMSCs.

## Figures and Tables

**Table 1:** Clinicopathological features of the samples analyzed by flow cytometry.

	BCC	SCC	Normal Skin
n (%)	118 (65.2%)	33 (18.2%)	30 (16.6%)
Age in years, mean (SD)	73.5 (±11.6)	84.6 (±7.8)	43.3 (±9.5)
F, n (%)/M, n (%)	55 (46.6%)/63 (53.4%)	13 (39.4%)/20 (60.6%)	19 (63.3%)/11 (36.7%)
Lesion location			
Head/neck, n (%)	102 (86.4%)	27 (82%)	1 (3.3%)
Trunk/limbs, n (%)	16 (13.6%)	6 (18%)	29 (96.7%)
Tumor subtype	Superficial = 13 (11%) Nodular = 41 (34.7%) Micronodular/infiltrative = 49 (41.5%) Other = 15 (12.7%)	Bowen = 5 (15.2%) Well differentiated = 14 (42.4%) Moderately/poorly differentiated = 9 (27.3%) Other = 5 (15.2%)	

BCC = basal cell carcinoma; SCC = squamous cell carcinoma; SD = standard deviation; F = female; M = male.

**Table 2:** Major CD3+ T cell subpopulations in NMSCs compared with normal skin (flow cytometry analysis—gating for CD3+ T cells).

	BCC (n = 111)	SCC (n = 33)	NS (n = 29)	*p*-Value †
CD3+CD45RO+ %, median (range)	98.9 (92.5–99.8)	98.8 (95.7–99.8)	93.7 (83.4–96.8)	BCC vs. NS, *p* < 0.001 *; SCC vs. NS, *p* < 0.001 *
CD4+ %, median (range)	57.0 (29.0–72.0)	55.0 (33.0–69.0)	58.0 (22.0–72.0)	BCC vs. SCC vs. NS, ns
CD8+ %, median (range)	22.0 (5.0–47.0)	25.0 (7.0–46.0)	23.0 (8.0–65.0)	BCC vs. SCC vs. NS, ns
CD4+/CD8+ ratio, median (range)	2.5 (0.6–14.3)	2.2 (0.8–10.3)	2.5 (0.3–8.6)	BCC vs. SCC vs. NS, ns
CD4+CD45RO+ %, median (range)	98.8 (47.7–99.9)	98.8 (95.8–99.9)	94.6 (87.9–96.8)	BCC vs. NS, *p* < 0.001 *; SCC vs. NS, *p* < 0.001 *
CD8+IFNγ+ Cells %, median (range)	52.3 (32.4–73.9)	56.5 (33.4–73.7)	32.1 (21.5–43.1)	BCC vs. NS, *p* < 0.001 *; SCC vs. NS, *p* < 0.001 *
γδ T Cells %, median (range)	1.0 (0.0–4.0)	1.0 (0.0–3.0)	2.0 (1.0–5.0)	BCC vs. NS, *p* = 0.002 *; SCC vs. NS, *p* = 0.001 *

BCC = basal cell carcinoma; SCC = squamous cell carcinoma; NS = normal skin; † Statistical significance was set to <0.017. (*) Statistically significant results; ns = differences not statistically significant.

**Table 3:** Major CD4+ T cell subpopulations in NMSCs compared with normal skin (flow cytometry analysis—gating for CD4+ T cells).

	BCC (n = 111)	SCC (n = 33)	NS (n = 29)	*p*-Value †
Th1 Cells %, median (range)	32.6 (15.1–48.2)	33.4 (19.4–49.7)	21.2 (9.3–37.8)	BCC vs. NS, *p* < 0.001 *; SCC vs. NS, *p* < 0.001 *
Th2 Cells %, median (range)	8.3 (2.4–15.6)	7.3 (3.7–13.1)	19.2 (9.4—30.9)	BCC vs. NS, *p* < 0.001 *; SCC vs. NS, *p* < 0.001 *
Th1/Th2 ratio, median (range)	3.7 (1.7–16.0)	4.6 (2.4–10.4)	1.1 (0.4–2.1)	BCC vs. NS, *p* < 0.001 *; SCC vs. NS, *p* < 0.001 *
Treg Cells %, median (range)	40.9 (28.3–54.0)	42.1 (26.8–56.7)	40.2 (14.0–56.3)	BCC vs. SCC vs. NS, ns
Th17 Cells %, median (range)	18.5 (1.9–57.9)	17.8 (3.5–55.0)	13.2 (4.1–31.8)	BCC vs. NS, *p* = 0.001 *; SCC vs. NS, *p* = 0.007 *
Th17/Treg %, median (range)	0.5 (0.1–1.3)	0.5 (0.1–1.3)	0.3 (0.2–0.7)	BCC vs. NS, *p* = 0.005 *. SCC vs. NS, ns
CD8/Treg %, median (range)	2.4 (0.4–11.3)	2.3 (0.4–10.5)	3.4 (0.8–32.5)	BCC vs. NS, *p* = 0.008 *; SCC vs. NS, *p* = 0.015 *

BCC = basal cell carcinoma; SCC = squamous cell carcinoma; NS = normal skin. † Statistical significance was set to <0.017. (*) Statistically significant results; ns = differences not statistically significant.

**Table 4:** Semi-quantitative assessment of T cells in BCCs, SCCs, and normal skin.

	BCC (n = 12)	SCC (n = 10)	NS (n = 10)	*p* Value †
CD3+, median (range)	2.2 (1.6–2.3)	2.2 (1.7–2.7)	0.6 (0.4–1.1)	BCC vs. NS *p* < 0.001 *; SCC vs. NS *p* < 0.001 *
CD4+, median (range)	2.0 (1.5–2.1)	2.0 (1.7–2.4)	0.6 (0.3–1.0)	BCC vs. NS *p* < 0.001 *; SCC vs. NS *p* < 0.001 *
CD8+, median (range)	1.7 (1.1–2.0)	1.7 (0.9–2.5)	0.3 (0.1–0.5)	BCC vs. NS *p* < 0.001 *; SCC vs. NS *p* < 0.001 *
FoxP3+, median (range)	1.0 (0.3–1.8)	1.3 (0.8–1.7)	0.1 (0.1–0.4)	BCC vs. NS *p* < 0.001 *; SCC vs. NS *p* < 0.001 *
CD8+/FoxP3+ Ratio (range)	1.6 (1.1–3.4)	1.3 (0.9–2.3)	1.5 (1.0–3.5)	BCC vs. SCC vs. NS, ns
CD3+ *P*, median (range)	2.9 (2.1–3.0)	2.8 (2.0–3.0)	-	-
CD3+ *In*, median (range)	0.3 (0.0–0.7)	0.5 (0.0–2.5)	-	-
CD4+ *P*, median (range)	2.7 (2.1–3.0)	2.7 (2.0–2.9)	-	-
CD4+ *In*, median (range)	0.3 (0.0–0.7)	0.4 (0.0–1.5)	-	-
CD8+ *P*, median (range)	2.3 (1.6–2.7)	2.3 (1.3–2.6)	-	-
CD8+ *In*, median (range)	0.0 (0.0–0.5)	0.3 (0.0–2.3)	-	-
FoxP3+ *P*, median (range)	1.4 (0.4–2.5)	1.7 (1.1–2.3)	-	-
FoxP3+ *In*, median (range)	0.0 (0.0–0.3)	0.0 (0.0–1.0)	-	-

BCC = basal cell carcinoma; SCC = squamous cell carcinoma; NS = normal skin; *P* = tumor periphery; *In* = inner area of the tumor. † Statistical significance was set to <0.017. (*) Statistically significant results; ns = differences not statistically significant.

## Data Availability

Data are contained within the article or Supplementary Materials.

## Supplementary Materials

The following supporting information can be downloaded at: https://www.mdpi.com/article/10.3390/cells13110964/s1. Table S1: Major CD3+ T cell subpopulations in BCCs compared with normal skin (flow cytometry analysis—gating for CD3+ T cells). Table S2: Major CD4+ T cell subpopulations in BCCs compared with normal skin (flow cytometry analysis—gating for CD4+ T cells). Table S3: Major CD3+ T cell subpopulations in SCCs compared with normal skin (flow cytometry analysis—gating for CD3+ T cells). Table S4: Major CD4+ T cell subpopulations in SCCs compared with normal skin (flow cytometry analysis—gating for CD4+ T cells). Table S5: Major CD3+ T cell subpopulations in SCCs compared with normal skin within the trunk and limbs (flow cytometry analysis—gating for CD3+ T cells). Table S6: Major CD4+ T cell subpopulations in SCCs compared with normal skin within the trunk and limbs (flow cytometry analysis—gating for CD4+ T cells).
