# Supplementary material for: Tumor-Infiltrating T Cells in Skin Basal Cell Carcinomas and Squamous Cell Carcinomas: Global Th1 Preponderance with Th17 Enrichment—A Cross-Sectional Study
Source: Cells. 2024 Jun 3;13(11):964. doi: 10.3390/cells13110964 (PMC11172364; doi:10.3390/cells13110964)
Supplement: Supplementary file 1 [file cells-13-00964-s001.zip › cells-3019940-supplementary.pdf]

cells MDPI

Supplementary data

Table S1. Major CD3+ T cell subpopulations in BCCs compared with Normal Skin (Flow Cytometry analysis – gating for CD3+ T cells)

	**BCC superficial** (n=13)	**BCC nodular** (n=41)	**BCC micronodular / infiltrative** (n=49)	**BCC other** (n=15)	**NS** (n=29)
CD3+CD45RO+ %, median (range) (BCC vs NS, p value†)	98.9 (92.6-99.6) *(p<0.001*)*	98.6 (92.5-99.8) *(p<0.001*)*	99.0 (94.4-99.7) *(p<0.001*)*	98.7 (97.1-99.7) *(p<0.001*)*	93.7 (83.4-96.8)
CD4+ %, median (range) (BCC vs NS, p value†)	57.0 (36.0-67.0) *(ns)*	59.0 (29.0-72.0) *(ns)*	56.0 (29.0-68.0) *(ns)*	56.0 (33.0-65.0) *(ns)*	58.0 (22.0-72.0)
CD8+ %, median (range) (BCC vs NS, p value†)	22.0 (5.0-40.0) *(ns)*	21.0 (7.0-46.0) *(ns)*	24.0 (8.0-47.0) *(ns)*	22.0 (6.0-46.0) *(ns)*	23.0 (8.0-65.0)
CD4+/CD8+, median (range) (BCC vs NS, p value†)	2.7 (0.9-14.3) *(ns)*	2.8 (0.6-9.9) *(ns)*	2.2 (0.6-8.0) *(ns)*	2.5 (0.9-10.3) *(ns)*	2.5 (0.3-8.6)
CD4+CD45RO+ %, median (range) (BCC vs NS, p value†)	98.8 (96.7-99.7) *(p<0.001*)*	98.6 (95.4-99.9) *(p<0.001*)*	98.9 (47.7-99.9) *(p<0.001*)*	99.0 (95.9-99.7) *(p<0.001*)*	94.5 (87.9-96.8)
CD8+IFNγ+ Cells %, median (range) (BCC vs NS, p value†)	56.0 (36.0-73.2) *(p<0.001*)*	56.5 (37.0-71.8) *(p<0.001*)*	48.0 (34.7-73.9) *(p<0.001*)*	53.7 (38.7-67.5) *(p<0.001*)*	32.3 (21.5-43.1)
γδ T Cells %, median (range) (BCC vs NS, p value†)	2.0 (0.0-3.0) *(ns)*	1.0 (0.0-3.0) *(p=0.001*)*	1.0 (0.0-4.0) *(p=0,013‡)*	1.5 (0.0-3.0) *(ns)*	2.0 (1.0-5.0)

BCC = basal cell carcinoma; NS = normal skin. Statistical significance was set to <0.013; (*) Statistically significant results; (‡) Marginally significant statistical results; ns = differences not statistically significant.

Table S2. Major CD4+ T cell subpopulations in BCCs compared with Normal Skin (Flow Cytometry analysis – gating for CD4+ T cells)

	**BCC superficial** (n=13)	**BCC nodular** (n=41)	**BCC micronodular / infiltrative** (n=49)	**BCC other** (n=15)	**NS** (n=29)
Th1 %, median (range) (BCC vs NS, p value†)	32.8 (23.4-42.7) *(p<0.001*)*	32.6 (18.7-48.2) *(p<0.001*)*	33.0 (15.1-43.2) *(p<0.001*)*	31.7 (20.0-39.8) *(p=0.001*)*	21.2 (9.3-37.8)
Th2 %, median (range) (BCC vs NS, p value†)	8.4 (7.0-15.6) *(p<0.001*)*	8.0 (4.1-14.4) *(p<0.001*)*	8.4 (2.4-14.9) *(p<0.001*)*	9.5 (6.1-14.6) *(p<0.001*)*	19.4 (9.4-30.9)
Th1/Th2, median (range) (BCC vs NS, p value†)	3.6 (2.1-4.8) *(p<0.001*)*	4.5 (1.9-8.3) *(p<0.001*)*	3.7 (1.7-16.0) *(p<0.001*)*	3.0 (2.1-6.4) *(p<0.001*)*	1.1 (0.4-2.1)

Treg %, median (range) (BCC vs NS, p value†)	41.2 (32.2-45.9) *(ns)*	40.3 (28.7-54.0) *(ns)*	40.6 (29.5-53.4) *(ns)*	40.5 (28.3-50.6) *(ns)*	39.5 (14.0-56.3)
Th17 %, median (range) (BCC vs NS, p value†)	19.9 (5.1-57.9) *(p=0.001*)*	16.6 (2.0-48.1) *(ns)*	17.7 (4.5-45.6) *(p=0.002*)*	18.8 (9.2-37.7) *(p=0.004*)*	13.1 (4.1-31.8)

BCC = basal cell carcinoma; NS = normal skin. Statistical significance was set to <0.013; (*) Statistically significant results; ns = differences not statistically significant.

Table S3. Major CD3+ T cell subpopulations in SCCs compared with Normal Skin (Flow Cytometry analysis - gating for CD3+ T cells)

	**SCC in situ** (n=5)	**SCC well-diff.** (n=14)	**SCC mod./poorly-diff.** (n=9)	**SCC other** (n=5)	**NS** (n=30)
CD3+CD45RO+ %, median (range) (SCC vs NS, p value†)	99.3 (96.1-99.8) *(p=0.001*)*	98.7 (95.7-99.5) *(p<0.001*)*	98.5 (96.8-99.3) *(p<0.001*)*	99.3 (96.7-99.5) *(p<0.001*)*	93.7 (83.4-96.8)
CD4 %, median (range) (SCC vs NS, p value†)	61.0 (50.0-68.0) *(ns)*	57.0 (40.0-69.0) *(ns)*	51.0 (40.0-65.0) *(ns)*	42.0 (33.0-66.0) *(ns)*	58.0 (22.0-72.0)
CD8 %, median (range) (SCC vs NS, p value†)	15.0 (7.0-30.0) *(ns)*	24.0 (7.0-46.0) *(ns)*	25.0 (14.0-43.0) *(ns)*	39.0 (15.0-46.0) *(ns)*	23.0 (8.0-65.0)
CD4/CD8, median (range) (SCC vs NS, p value†)	4.0 (1.7-10.3) *(ns)*	2.3 (0.9-9.3) *(ns)*	2.2 (1.0-4.6) *(ns)*	1.1 (0.8-4.4) *(ns)*	2.5 (0.3-8.6)
CD4+CD45RO+ %, median (range) (SCC vs NS, p value†)	99.5 (95.9-99.7) *(p=0.001*)*	98.3 (95.8-99.7) *(p<0.001*)*	98.9 (97.3-99.9) *(p<0.001*)*	98.4 (96.8-99.2) *(p<0.001*)*	94.6 (87.9-96.8)
CD8+IFNγ+ Cells %, median (range) (SCC vs NS, p value†)	47.3 (41.9-68.5) *(p=0.001*)*	57.3 (33.4-69.7) *(p<0.001*)*	58.6 (50.3-73.7) *(p<0.001*)*	60.9 (54.2-62.1) *(p<0.001*)*	32.1 (21.5-43.1)
γδ T Cells %, median (range) (SCC vs NS, p value†)	2.0 (1.0-2.0) *(ns)*	1.0 (1.0-3.0) *(ns)*	1.0 (0.0-1.0) *(p=0.001*)*	1.0 (1.0-2.0) *(ns)*	2.0 (1.0-5.0)

SCC = squamous cell carcinoma; NS = normal skin; SCC well-diff. = well-differentiated SCC; SCC mod./poorly-diff. = moderately to poorly-differentiated SCC. Statistical significance was set to <0.013; (*) Statistically significant results; ns = differences not statistically significant.

Table S4. Major CD4+ T cell subpopulations in SCCs compared with Normal Skin (Flow Cytometry analysis – gating for CD4+ T cells)

	**SCC in situ** (n=5)	**SCC well-diff.** (n=14)	**SCC mod./poorly-diff.** (n=9)	**SCC other** (n=5)	**NS** (n=30)
Th1 %, median (range)<br>(SCC vs NS, p value†)	33.8 (26.4-36.9)<br>*(ns)*	30.2 (22.7-45.7)<br>*(p=0.001*)*	30.5 (24.6-47.9)<br>*(p=0.003*)*	35.7 (19.4-49.7)<br>*(p=0.007*)*	21.1 (9.3-37.8)
Th2 %, median (range)<br>(SCC vs NS, p value†)	8.1 (4.4-10.3)<br>*(p<0.001*)*	7.5 (3.7-13.1)<br>*(p<0.001*)*	8.8 (6.2-12.6)<br>*(p<0.001*)*	6.1 (4.7-9.6)<br>*(p<0.001*)*	19.2 (9.4-30.9)
Th1/Th2, median (range)<br>(SCC vs NS, p value†)	4.2 (2.6-7.8)<br>*(p<0.001*)*	4.7 (2.5-10.4)<br>*(p<0.001*)*	4.7 (2.4-5.3)<br>*(p<0.001*)*	5.7 (4.1-7.9)<br>*(p<0.001*)*	1.1 (0.4-2.1)
Treg %, median (range)<br>(SCC vs NS, p value†)	40.7 (30.8-51.7)<br>*(ns)*	42.6 (35.8-56.7)<br>*(ns)*	41.9 (31.7-50.3)<br>*(ns)*	35.3 (26.8-51.9)<br>*(ns)*	40.2 (14.0-56.3)
Th17 %, median (range)<br>(SCC vs NS, p value†)	16.4 (7.7-26.9)<br>*(ns)*	17.0 (3.5-34.6)<br>*(ns)*	27.6 (6.7-55.0)<br>*(p=0.003*)*	16.5 (5.5-20.0)<br>*(ns)*	13.2 (4.1-31.8)

SCC = squamous cell carcinoma; NS = normal skin; SCC well-diff. = well-differentiated SCC; SCC mod./poorly-diff. = moderately to poorly-differentiated SCC. Statistical significance was set to <0.013; (*) Statistically significant results; ns = differences not statistically significant.

Table S5. Major CD3+ T cell subpopulations in SCCs compared with Normal Skin within Trunk and Limbs (Flow Cytometry analysis – gating for CD3+ T cells)

	**BCC** (n=16)	**SCC** (n=6)	**NS** (n=28)	**p value†**
CD3+CD45RO+ %, median (range)	98.8 (92.6-99.6)	99.1 (97.4-99.8)	93.7 (83.4-96.8)	BCC vs NS, p<0.001*; SCC vs NS, p<0.001*
CD4+ %, median (range)	57.0 (36.0-66.0)	60.0 (44.0-68.0)	58.0 (22.0-72.0)	BCC vs SCC vs NS, ns
CD8+ %, median (range)	22.0 (13.0-40.0)	17.0 (7.0-38.0)	23.0 (8.0-65.0)	BCC vs SCC vs NS, ns
CD4+/CD8+ ratio, median (range)	2.5 (0.9-5.1)	3.8 (1.2-10.3)	2.5 (0.3-8.6)	BCC vs SCC vs NS, ns
CD4+CD45RO+ %, median (range)	99.0 (96.7-99.7)	99.3 (95.9-99.7)	94.5 (87.9-96.8)	BCC vs NS, p<0.001*; SCC vs NS, p<0.001*
CD8+IFNγ+ Cells %, median (range)	55.5 (36.0-64.8)	52.4 (41.9-68.5)	31.9 (21.4-43.1)	BCC vs NS, p<0.001*; SCC vs NS, p=0.006*
γδ T Cells %, median (range)	1.0 (0.0-0.3)	1.5 (1.0-2.0)	2.0 (1.0-5.0)	BCC vs SCC vs NS, ns

BCC = basal cell carcinoma; SCC = squamous cell carcinoma; NS = normal skin. Statistical significance was set to <0.017; (*) Statistically significant results; ns = differences not statistically significant.

Table S6. Major CD4+ T cell subpopulations in SCCs compared with Normal Skin within Trunk and Limbs (Flow Cytometry analysis – gating for CD4+ T cells)

	**BCC** (n=16)	**SCC** (n=6)	**NS** (n=28)	**p value†**
Th1 Cells %, median (range)	32.9 (22.0 46.0)	30.0 (25.4-36.9)	21.0 (9.3-37.8)	BCC vs NS, p<0.001*; SCC vs NS, p=0.009*
Th2 Cells %, median (range)	8.5 (4.5-14.6)	8.2 (3.9-10.3)	19.4 (9.4-30.9)	BCC vs NS, p<0.001*; SCC vs NS, p<0.001*
Th1/Th2 ratio, median (range)	3.9 (2.2-6.5)	4.3 (2.6-7.6)	1.1 (0.4-2.1)	BCC vs NS, p<0.001*; SCC vs NS, p=0.01*
Treg Cells %, median (range)	42.6 (28.3-52.8)	41.7 (30.8-51.1)	39.5 (14.0-56.3)	BCC vs SCC vs NS, ns
Th17 Cells %, median (range)	19.8 (5.1-35.8)	10.8 (3.5-26.9)	13.1 (4.1-22.3)	BCC vs NS, p<0.003*; SCC vs NS, ns
Th17/Treg %, median (range)	0.5 (0.2-0.9)	0.3 (0.1-0.7)	0.3 (0.2-0.6)	BCC vs SCC vs NS, ns
CD8/Treg %, median (range)	2.4 (1.6-8.0)	1.5 (0.4-5.4)	3.4 (0.8-32.5)	BCC vs SCC vs NS, ns

BCC = basal cell carcinoma; SCC = squamous cell carcinoma; NS = normal skin. Statistical significance was set to <0.017; (*) Statistically significant results; ns = differences not statistically significant.
